# Parasite-infected sticklebacks increase the risk-taking behaviour of uninfected group members

**DOI:** 10.1098/rspb.2018.0956

**Published:** 2018-06-20

**Authors:** Nicolle Demandt, Benedikt Saus, Ralf H. J. M. Kurvers, Jens Krause, Joachim Kurtz, Jörn Peter Scharsack

**Affiliations:** 1 Institute for Evolution and Biodiversity, University of Münster, Hüfferstrasse 1, 48149 Münster, Germany; 2 Center for Adaptive Rationality, Max Planck Institute for Human Development, Lentzeallee 94, 14195 Berlin, Germany; 3 Leibniz-Institute of Freshwater Ecology and Inland Fisheries, Müggelseedamm 310, Berlin, Germany; 4 Faculty of Life Sciences Humboldt-Universität zu Berlin, Invalidenstrasse 42, 10115 Berlin, Germany

**Keywords:** *Gasterosteus aculeatus*, *Schistocephalus solidus*, behavioural manipulation, group behaviour, risk-taking behaviour, quorum decision

## Abstract

Trophically transmitted parasites frequently increase their hosts' risk-taking behaviour, to facilitate transmission to the next host. Whether such elevated risk-taking can spill over to uninfected group members is, however, unknown. To investigate this, we confronted groups of 6 three-spined sticklebacks, *Gasterosteus aculeatus*, containing 0, 2, 4 or 6 experimentally infected individuals with a simulated bird attack and studied their risk-taking behaviour. As a parasite, we used the tapeworm *Schistocephalus solidus*, which increases the risk-taking of infected sticklebacks, to facilitate transmission to its final host, most often piscivorous birds. Before the attack, infected and uninfected individuals did not differ in their risk-taking. However, after the attack, individuals in groups with only infected members showed lower escape responses and higher risk-taking than individuals from groups with only uninfected members. Importantly, uninfected individuals adjusted their risk-taking behaviour to the number of infected group members, taking more risk with an increasing number of infected group members. Infected individuals, however, did not adjust their risk-taking to the number of uninfected group members. Our results show that behavioural manipulation by parasites does not only affect the infected host, but also uninfected group members, shedding new light on the social dynamics involved in host–parasite interactions.

## 1. Introduction

Many parasites manipulate behaviours of their host [1–3]. Particularly, parasites with complex life cycles, involving more than one host species, have evolved strategies to manipulate host behaviour to facilitate transmission to the next host [3–6]. A prominent example is *Toxoplasma gondii*, a felid parasite which encysts in the brain of its natural intermediate rodent hosts, mice and rats, and changes their innate aversion to cats into imprudent attraction [7,8]. Another example is the tapeworm *Ligula intestinalis*, infected roach (*Rutilus rutilus*) swim closer to shore, to facilitate transmission to its final bird hosts [9].

An important consequence of complex life cycles is that these parasites generally do not transmit directly between individuals of the same host species. Thus, interactions between uninfected and infected conspecifics do not directly increase the infection risk of the uninfected individuals. Nevertheless, the presence of infected individuals in social groups may still be costly for uninfected conspecifics, when information flows between group members generate collective responses [10,11].

The presence of a threshold number of infected individuals within a group might influence the decision-making process of the whole group, because quorum responses are often involved when making collective movement decisions, both in the presence and absence of predators [12–17]. Accordingly, if an infection alters the behaviour of the infected individual, this might influence the behaviour of uninfected group members and the group as a whole. Increased risk-taking behaviour of infected individuals might, therefore, also affect behaviours of other group members.

To address this question, we used three-spined sticklebacks (*Gasterosteus aculeatus*) and their infection with the tapeworm *Schistocephalus solidus*, an important model in ecological and evolutionary parasitology [18,19]. The three-spined stickleback is a small teleost fish that occurs in coastal marine and freshwater habitats all over the Northern Hemisphere [20]. The anti-predator behaviour of sticklebacks covaries with predation risk, so that sticklebacks display more anti-predatory behaviour in predator-rich environments [21]. To reduce the risk of detection by a predator, sticklebacks generally avoid open areas and stay near vegetation or other forms of cover [21]. Once a predator is encountered, sticklebacks can form large shoals, up to several hundred individuals, to minimize individual predation risk [21,22].

The tapeworm *S. solidus* is a frequent parasite in sticklebacks [20]. *Schistocephalus solidus* reproduces in the gut of its final bird host. The eggs are then passed with the bird's faeces into water where the larvae hatch and infect the first intermediate host, a cyclopoid copepod [19]. The infected copepods are then ingested by the second, specific and obligatory host of *S. solidus*, the three-spined stickleback [18,23]. In the stickleback, *S. solidus* penetrates the gut wall and grows substantially in the body cavity and reaches in extreme cases up to 50% of the fish's body weight [24].

*Schistocephalus solidus* infections can cause distinct changes in the anti-predator behaviour of individual sticklebacks [5,25]. First, *S. solidus* infection reduces the escape response of its stickleback host to predators and increases their risk-taking behaviour in dangerous feeding situations, thus facilitating the parasite's transmission to its final host, most often piscivorous birds [26–29]. Second, *S. solidus* infection reduces sociality of sticklebacks [30]. One proposed explanation for this is that infection reduces competitive foraging ability, forcing infected individuals to move away from conspecifics to reduce feeding competition [30].

However, even when satiated, *S. solidus-*infected sticklebacks spend less time shoaling than uninfected sticklebacks [30]. Separation of infected sticklebacks from the shoal might also increase the parasite's likelihood of transmission to the next host [30].

The observation that *S. solidus-*infected sticklebacks decrease social responsiveness to conspecifics and increase risk-taking, in turn, may have consequences for the behaviour of nearby uninfected sticklebacks. To investigate this, we studied groups of six sticklebacks with either 0, 2, 4 or 6 *S. solidus*-infected conspecifics. We determined the risk-taking behaviour of each shoal member before and after a simulated bird attack. We expected that uninfected sticklebacks are susceptible to the presence of infected, behaviourally altered conspecifics in their shoal. Conversely, *S. solidus*-infected sticklebacks were predicted to be unaffected by the behaviour of uninfected conspecifics.

## 2. Material and methods

### (a) Experimental animals

Laboratory-bred F_1_ offspring of wild-caught three-spined sticklebacks and *S. solidus* parasites, collected in April 2016 at the brook Ibbenbürener Aa (Germany, 52°17′33.51″ N 7°36′45.46″ E), were used. F_1_ families were obtained by *in vitro* fertilization and housed in family groups in 16 l tanks (VewaTech, Germany) with artificial plants as shelter. Sticklebacks were maintained in recirculating tap water at 18°C with a constant 16 L : 8 D cycle and fed daily ad libitum with frozen *Chironomid* larvae. Four weeks before the start of the experiment, dry food flakes (Tetra, Germany) were added to the diet to familiarize the sticklebacks with the food stimulus used during the experimental trials.

For parasite reproduction, the tapeworms were bred *in vitro* [31,32] in size-matched pairs to increase the probability of outcrossing [33]. Parasite eggs were washed and stored at least two weeks at 4°C to simulate winter conditions. The eggs were then incubated for three weeks at 20°C in the dark to enable coracidia (i.e. tapeworm larvae) development. The hatching of coracidia was initiated subsequently by illumination and eggs were kept in a 16 L : 8 D cycle for 2 more days. Hatched coracidia (1–8) were transferred to individual copepods in wells of 24 well plates with 2 ml tap water. Fourteen days post-exposure, the copepods were checked with a microscope for *S. solidus* infection.

At three months of age, the experimental sticklebacks (*n* = 324) were taken from nine families and placed in 18 groups of 18 fish, each group containing two fish from each family, in 14 l home tanks. Two months later, after being starved for 2 days and transferred into individuals jars with 400 ml tank water, the sticklebacks were either offered *S. solidus-*infected copepods (*n* = 216) or uninfected copepods (*n* = 108). After 69 days, the presence of *S. solidus* plerocercoids in the sticklebacks' body cavity was determined by inspecting the swelling of the body of all exposed sticklebacks [34]; 85 out of 216 exposed sticklebacks were determined to be successfully infected (which was further confirmed by direct measurement of parasite burden after the experiment, see below).

### (b) Experimental set-up

To investigate the effects of shoal composition on the risk-taking behaviour of infected and uninfected sticklebacks before and after an artificial bird attack, four treatment groups were created: six uninfected sticklebacks (6u), four uninfected and two infected sticklebacks (4u/2i), two uninfected and four infected sticklebacks (2u/4i) and six infected sticklebacks (6i). All uninfected sticklebacks were taken from the group sham exposed to the parasite. All treatment groups were replicated seven times resulting in a total of 84 infected and 84 uninfected sticklebacks.

**Figure 1.**
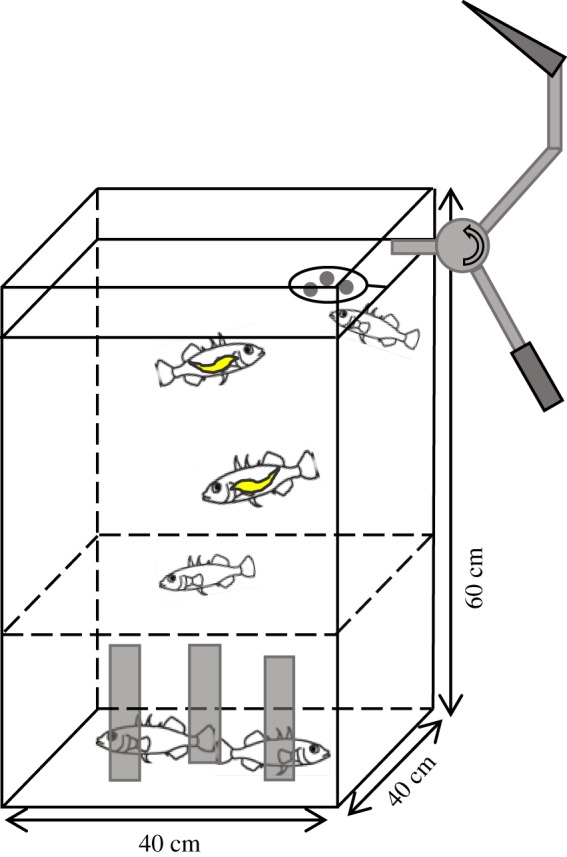
Scheme of the experimental tank. The tank contained artificial plants (grey rectangles) providing shelter at the bottom of the tank (‘safe’ zone). The dashed line above the shelters indicates the boundary to the ‘dangerous’ open water zone. At the water surface, a food stimulus was provided in a floating ring and an artificial beak was used to simulate a bird attack. (Online version in colour.)

The experimental tank (40 × 40 × 60 cm) was divided into two vertical zones (figure 1): a ‘safe’ zone at the bottom with three artificial plants providing shelter and a ‘dangerous’ upper zone without shelter and including the surface, where the artificial bird attack was triggered (see below). A transparent floating ring was positioned under a funnel to provide the food stimulus and an artificial bird beak was attached to the experimental tank. The bird beak was unobservable for the sticklebacks when not triggered [35]. Opaque Plexiglas covered three walls and the bottom of the tank. On the open side of the tank, a Logitech HD pro c 920 webcam was placed to record the trials and a Canon EOS 5D mark II camera took high-resolution photos every minute. The complete set-up was shielded by black cloths, from behind which the operator provided the food stimuli and triggered the bird attack.

### (c) Test procedure and behavioural observation

Sticklebacks were not fed on the day of testing in order to increase their motivation to feed. For each trial, the required number of infected and/or uninfected sticklebacks was randomly taken from six home tanks, assuring that all sticklebacks within a shoal were unfamiliar to each other. Fish were put simultaneously into the experimental tank and given a 30 min acclimatization period, before the observation started. After 10 min of observation (i.e. before the bird attack), a food stimulus was provided in the floating ring. When at least five sticklebacks had approached the water surface (i.e. within two body lengths), an artificial bird attack was triggered and a second food stimulus was provided in the floating ring, to stimulate the sticklebacks to re-enter the ‘dangerous’ zone. Behavioural observations continued for another 10 min (after the bird attack). Then the sticklebacks were collected and transferred to a new home tank. The experimental tank was cleaned and filled with new water between trials.

The behaviour of each individual was analysed using the photo and video recordings. The videos were analysed with VLC player. A mask marking the border between the ‘safe’ and ‘dangerous’ zone was placed on the video screen. For each shoal, every individual was marked on the first photo and the video was started when the individual was in the same position as on the photo. The infection status of each individual was determined by examining the swelling of the body cavity [34] on the high-resolution photographs. For each stickleback, the time spent in the ‘dangerous’ zone was recorded 10 min before the bird attack. Once the bird attack was triggered, it was recorded for each fish whether it escaped to the ‘safe’ zone or remained in the ‘dangerous’ zone. After all six sticklebacks had stopped their escape behaviour (i.e. when they stopped their rapid downward movement and resumed swimming at slow speed), the time each stickleback spent in the ‘dangerous zone’ was recorded for another 10 min.

### (d) Statistical analysis

For statistical analyses R v. 3.3.2 [36] was used. The data were tested in four subsets: ‘pure groups’, comprising the shoals with all uninfected (6u) and all infected (6i) individuals; ‘uninfected’ sticklebacks, to test whether uninfected individuals were affected by the abundance of infected shoal members; ‘infected’ to test whether *S. solidus-*infected individuals were affected by the abundance of uninfected shoal members and ‘mixed groups’, comprising uninfected and infected sticklebacks (4u/2i and 2u/4i) to test for an interaction between individual and shoal infection status.

Visual inspection of the data suggested a possible need to model a variance structure for all models. Three different variance structures (i.e. group, individual and individual nested within group) were compared to determine the optimal variance structure for all models. This was done with a parametric bootstrap ‘PBmodcomp’ (package ‘pbkrtest’ [37]) test, and comparison of the Akaike information criterion (AIC) values (package ‘stats’ [36]). Both the bootstrap and the AIC values indicated that the models containing only the intercept for group were the best fit, so this structure was used for further analyses in all but one model (see below for details).

The residuals of all selected models were visually inspected for normality and homogeneity. In the case of non-normality of the residuals, another model that met the assumptions of the model was used for further analysis, which occurred only for the time spent in the ‘dangerous’ zone for the ‘pure groups’. In this case, a random intercept for group number and a random slope for treatment group were used. The fixed effect parameters with function ‘fixef’ (package ‘nlme’ [38]) were used to inspect all generalized linear mixed models (GLMMs) for possible over-fitting.

To analyse the effect of individual infection status and treatment group on the likelihood of remaining in the ‘dangerous’ versus escaping to the ‘safe’ zone after the bird attack, we used a GLMM with the function ‘glmer’ (package ‘lme4’ [39]) with a binomial distribution. Treatment group was fitted as a fixed effect in all subsets. For the ‘mixed groups’, we additionally fitted as fixed effects individual infection status and its interaction with treatment group.

To analyse the effect of individual infection status, treatment group and bird attack on time spent in the ‘dangerous’ zone, we used linear mixed models with the function ‘lmer’ (package ‘lme4’ [39]). Treatment group, time before or after the bird attack and their interaction were fitted as fixed effects in all four subsets. For the ‘mixed groups’, we additionally fitted as fixed effects individual infection status, the two-way interactions between infection status and treatment group, the two-way interaction between infection status and time before or after the bird attack and the three-way interaction between individual infection status, treatment group and time before or after the bird attack.

All full models were refitted to maximum likelihood, after which a parametric bootstrapping with function ‘PBmodcomp’ (package ‘pbkrtest’ [37]) was used to find the minimum adequate model (MaM) by manually testing all interactions and fixed effects. All parametric bootstraps were run with 10 000 simulations, using the function ‘makeCluster’ (package ‘parallel’, [36]), in combination with the function ‘detectCores’ (package ‘parallel’, [36]). After finding the MaM, all models were refitted to restricted maximum likelihood, *post hoc* tests were performed with only the function ‘glht’ (package ‘multcomp’ [40]), when no interactions were significant, and in combination with the function ‘lsm’ (package ‘lsmeans’ [41]) for pairwise comparison, whenever an interaction was significant.

### (e) Quorum decision-making

To investigate whether potential behavioural responses of uninfected sticklebacks to the number of infected shoal members followed linear or nonlinear responses, we studied quorum decision-making. In a quorum response, the probability that an individual shows a particular behaviour increases in a step-like manner with the number of other individuals showing a particular behaviour. We used the following equation [15,42,43]:2.4
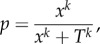


where *p* is the probability that a focal individual chooses a particular option (here, the response of uninfected individuals to the bird attack: escape zone and time spent in ‘dangerous’ zone after bird attack), *x* is the number of individuals that have already chosen this option (here, the number of infected shoal members), *T* is the threshold quorum at which the response has the steepest increase and *k* determines the steepness of this increase. A quorum response occurs if *k* ≥ 2 [15,42,43] with higher values implying stronger quorum responses. The *T* and *k* values of uninfected individuals for both the escape behaviour and the time spent in the ‘dangerous’ zone were calculated using the function ‘nls’ (package ‘stats’ [36]) in R.

### (f) Parasite burden

To confirm infections, all individuals labelled as ‘infected’ were dissected two months after the behavioural trials. They were weighed (to the nearest mg), killed by decapitation, dissected and screened for parasites. Parasites were weighed (to the nearest milligrams) and the parasite burden was calculated as per cent parasite weight of total weight (i.e. stickleback weight plus parasite weight). A generalized linear model with a gamma distribution and the function ‘glm’ (package ‘stats’ [36]) were used to test for a difference in parasite burden between treatment groups.

The average number of parasites per infected stickleback was 1.5 ± 1.06, resulting in an average parasite burden of 25 ± 7% w/w. There were no differences in parasite burden between the infected sticklebacks of the different treatment groups (*χ*^2^ = 2.396, d.f. = 2, *p* = 0.3).

## 3. Results

### (a) Effects of parasite infection on escape behaviour

**Figure 2.**
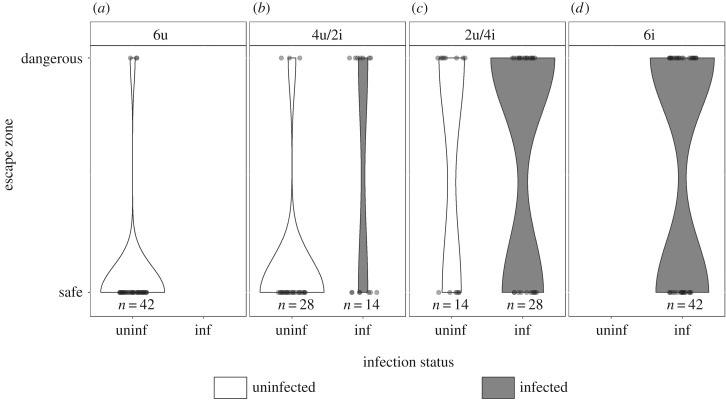
Escape zones (‘safe’ versus ‘dangerous’) used by sticklebacks after the bird attack. The violin graphs are split by the four treatment groups: (*a*) 6u, (*b*) 4u/2i, (*c*) 2u/4i or (*d*) 6i and results are shown separately for uninfected (white) and *S. solidus*-infected (grey) individuals. The shape of the violins is scaled proportionally to the number of sticklebacks that fled to the ‘safe’ or remained in the ‘dangerous’ zone.

To study the escape behaviour of uninfected and *S. solidus*-infected sticklebacks after an artificial bird strike, we recorded for each individual if it escaped to the ‘safe’ zone or remained in the ‘dangerous’ zone. In the ‘pure groups’ comprising only uninfected and only infected sticklebacks, uninfected (u) sticklebacks were more likely to escape into the ‘safe’ zone after the bird attack (6u: 93% ‘safe’ zone), than infected (i) fish (6i: 55% ‘safe’ zone) (*χ*^2^ = 12.841, d.f. = 1, *p* = 0.007; figure 2*a*,*d*).

Comparing across all groups, the escape behaviour of infected sticklebacks was not influenced by the number of uninfected shoal members (*χ*^2^ = 0.377, d.f. = 2, *p* = 0.82; figure 2*b*–*d*), but uninfected sticklebacks changed their escape behaviour according to the number of infected shoal members (*χ*^2^ = 8.282, d.f. = 2, *p* = 0.037; figure 2*a*–*c*). *Post hoc* tests revealed that uninfected sticklebacks in the treatment group 2u/4i remained more often in the ‘dangerous’ zone compared with the uninfected sticklebacks in treatment groups 6u (*Z* = 2.544, *p* = 0.03) and 4u/2i (*Z* = 2.294, *p* = 0.06; figure 2*a*–*c*). Uninfected sticklebacks in the treatment groups 6u and 4u/2i did not differ (*Z* = −0.011, *p* = 1.000; figure 2*a*,*b*).

In the ‘mixed groups’ 2u/4i and 4u/2i, a significant interaction between infection status and treatment group was detected (*χ*^2^ = 6.6805, d.f. = 1, *p* = 0.02; figure 2*b*,*c*). In groups with a minority of infected sticklebacks (4u/2i), uninfected sticklebacks more often escaped to the ‘safe’ zone compared to infected ones (*Z* = 2.812, *p* = 0.02; figure 2*b*). By contrast, in groups with a majority of infected sticklebacks (2u/4i), there was no difference in escape behaviour between infected and uninfected fish (*Z* = 0.302, *p* = 0.99).

### (b) Effect of parasite infection on risk-taking behaviour

**Figure 3.**
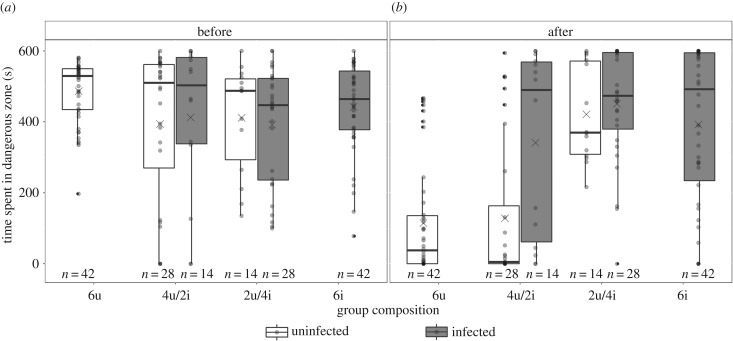
Time spent in the ‘dangerous’ zone (*a*) before and (*b*) after a simulated bird attack. Results are shown per treatment group: 6u, 4u/2i, 2u/4i and 6i; and for uninfected (white) and *S. solidus*-infected (grey) individuals. The edges of the boxplots indicate the first and third quartiles, the solid lines the median, the crosses the mean, the whiskers the highest and lowest values within 1.5-fold of the inter-quartile range and the dots, the outliers.

To investigate whether *S. solidus*-infected sticklebacks influence the risk-taking behaviour of sticklebacks in shoals with different numbers of infected sticklebacks, we looked at the time individuals spend in the ‘dangerous’ zone of the test tank, before and after an artificial bird attack. In the ‘pure groups’ with either ‘all uninfected’ or ‘all infected’ sticklebacks, the time spent in the ‘dangerous’ zone was significantly influenced by the interaction between the simulated bird attack and treatment group (*χ*^2^ = 48.462, d.f. = 1, *p* < 0.001; figure 3*a*,*b*). Before the bird attack, uninfected and infected individuals from these groups did not differ in their time spent in the ‘dangerous’ zone (*t* = 0.875, *p* = 0.81; figure 3*a*). After the bird attack, individuals from ‘all infected’ groups spent more time in the ‘dangerous’ zone than individuals from ‘all uninfected’ groups (6u) (*t* = −5.487, *p* < 0.001; figure 3*b*). Moreover, individuals from ‘all infected’ groups did not change their time spent in the ‘dangerous’ before and after the bird attack (*t* = −1.634, *p* = 0.35; figure 3*a*,*b*), whereas individuals from ‘all uninfected’ groups spent significantly less time in the ‘dangerous’ zone after the bird attack (*t* = −12.237, *p* < 0.001; figure 3*a*,*b*).

Comparing the time spent in the ‘dangerous’ zone for uninfected sticklebacks across all groups, revealed a significant interaction between the bird attack and the number of infected sticklebacks in the group (*χ*^2^ = 41.163, d.f. = 2, *p* < 0.001; figure 3*a*,*b*). Before the bird attack, the time uninfected individuals spent in the ‘dangerous’ zone groups did not differ across groups (all *p* > 0.7; figure 3*a*). After the bird attack, however, the time uninfected individuals spent in the ‘dangerous’ zone differed between groups: uninfected sticklebacks in the treatment group 2u/4i spent more time in the ‘dangerous’ zone than uninfected sticklebacks in the treatment groups 6u (*t* = −13.287, *p* < 0.001) and 4u/2i (*t* = −7.757, *p* < 0.001; figure 3*b*).

Infected individuals showed a non-significant trend for an interaction between bird attack and the number of uninfected group members (*χ*^2^ = 5.071, d.f. = 2, *p* = 0.09; figure 3*a*, *b*). However, their time in the ‘dangerous’ zone was neither significantly influenced by the bird attack (*χ*^2^ = 0.487, d.f. = 1, *p* = 0.50; figure 3*a*,*b*), nor by the number of uninfected group members (*χ*^2^ = 0.517, d.f. = 2, *p* = 0.80; figure 3*a*,*b*).

In the ‘mixed groups’ (4u/2i and 2u/4i), the time spent in the ‘dangerous’ zone showed a significant three-way interaction between treatment group, infection status and bird attack (*χ*^2^ = 19.472, d.f. = 1, *p* < 0.001; figure 3*a*,*b*). Before the bird attack there was again no difference in the time uninfected and infected sticklebacks in both treatment groups spent in the ‘dangerous’ zone (4u/2i: *t* = −0.491, *p* = 1 and 2u/4i: 2.093, *p* = 0.31; figure 3*a*). After the bird attack, uninfected sticklebacks in the groups with more uninfected individuals (4u/2i), spent significantly less time in the ‘dangerous’ zone than their infected group members (*t* = −4.919, *p* < 0.001; figure 3*b*). By contrast, in the groups with more infected individuals (2u/4i), uninfected sticklebacks spent similar amounts of time in the ‘dangerous’ zone after the bird attack as their infected group members (*t* = −0.697, *p* = 0.98; figure 3*b*).

### (c) Sticklebacks adjust their behaviour to infected conspecifics with quorum decisions

Uninfected sticklebacks thus adjusted their escape behaviour (figure 2) and their time spent in the ‘dangerous’ zone (figure 3) to the behaviour of *S. solidus*-infected group members. This ‘decision’ of uninfected sticklebacks was based on the number of infected group members and it was tested whether uninfected sticklebacks responded in a quorum-like manner. The estimated *k* value of 3.5 for escape behaviour and 3.1 for time spent in the ‘dangerous’ zone after the bird attack suggest that uninfected sticklebacks indeed used quorum responses rather than linear responses when adjusting their behaviour to that of infected conspecifics. In sum, uninfected sticklebacks appear to behave like infected ones above a certain threshold number of infected fish in the shoal.

## 4. Discussion

Although many parasites alter the anti-predator behaviour of their hosts to facilitate transmission to the next host, effects of such behavioural changes on the hosts' social environment have received far less attention. Here, we tested how sticklebacks, behaviourally altered by infections with the tapeworm *S. solidus*, influenced the escape and risk-taking behaviour of uninfected group members after a simulated bird attack. Strikingly, when the infected sticklebacks within a group outnumbered the uninfected sticklebacks, the uninfected sticklebacks changed their response to the bird attack and behaved like *S. solidus-*infected sticklebacks. In these shoals, uninfected sticklebacks did not escape to the ‘safe’ zone as frequently and remained in the ‘dangerous’ zone longer. Uninfected sticklebacks adjusted their behaviour in a quorum-like manner to the risk-taking behaviour of *S. solidus-*infected sticklebacks. The decisions of uninfected sticklebacks to follow their infected conspecifics, may be an attempt to maintain shoal cohesion rather than a motivation for higher risk-taking, even though the latter was the consequence of their behavioural response.

The adjustment of the risk-taking behaviour of uninfected sticklebacks based on a quorum threshold is presumably originally an anti-predatory strategy as it increases the likelihood of the uninfected sticklebacks to join the largest shoal. Ward *et al*. [12,13] showed that quorum responses to conspecifics explained sticklebacks' collective movement decisions in the context of predator avoidance and food patch detection. The authors suggested that a quorum threshold of two individuals has probably evolved because it is rare that two individuals make the same mistake (e.g. of approaching a ‘dangerous’ predator) at the same time [12,13].

Quorum decision-making can contribute to higher group cohesion [42], higher decision accuracy [15,42–46] and faster decision-making [42], all of which might contribute to a higher confusion effect in the presence of a predator [47]. Even when the shoal makes a wrong decision, staying together might still be the more beneficial option, as the chance of an individual fish to be captured by a predator may increase when leaving the shoal [47,48]. In this study, infected behaviourally manipulated individuals made decisions, and the consequential quorum decision of uninfected specimen, to follow the group pressure, was arguably wrong. In humans, it has long been established that wrong decisions of individuals are triggered more often if a majority of group members displays erroneous decisions [49]. However, humans use social information to adjust their quorum threshold [45] and if the wrong decisions were displayed by a group of robot peers, test persons did not conform to the ‘peer pressure’, suggesting that they evaluated the quality of the source of information [50]. In this study, uninfected sticklebacks might have been unable to sense the infection status of their conspecifics and failed to readjust their quorum threshold accordingly.

In the wild, the prevalence (% infected sticklebacks) of *S. solidus* infections varies substantially across seasons, years and habitats [24]. Long-term monitoring of *S. solidus* prevalence revealed habitats with consistently low prevalence of 1–3% or even less and others with consistently high (greater than 50%) prevalence of the parasite [24,51–53]. Accordingly, it would be interesting to compare quorum thresholds of population of sticklebacks varying in parasite prevalence (but similar predation pressure) to test whether parasite prevalence covaries with quorum thresholds.

Contrary to uninfected sticklebacks, the *S. solidus-*infected ones did not adjust their risk-taking behaviour to the group composition. We propose two possible, non-mutually exclusive, explanations for this result. First, the trade-off between shoaling and foraging [47] might differ between *S. solidus-*infected and uninfected sticklebacks. This is likely because *S. solidus-*infected sticklebacks have a higher energy requirement [18,54] and due to the distension of their stomach, *S. solidus-*infected sticklebacks also need to consume prey more frequently than uninfected sticklebacks [26]. Additionally, the energetic demands caused by the parasites also increase the resting metabolism of the infected sticklebacks, which makes swimming even more costly for them [55]. Taken together, a higher metabolism and urge to forage, might explain why the *S. solidus-*infected sticklebacks did not seem to be influenced by the number of uninfected sticklebacks within their shoal.

Second, an *S. solidus* infection may directly influence the social behaviour of the infected host. Barber *et al*. [30] showed that an *S. solidus* infection also influences the shoaling behaviour of infected sticklebacks, so that *S. solidus-*infected sticklebacks prefer positions outside the shoal and spend less time near a shoal than their uninfected conspecifics. The alterations in shoaling behaviour might make the infected sticklebacks less susceptible to the influences of their uninfected conspecifics, while at the same time making them more susceptible to predators. Similarly, Barber & Huntingford [56] observed that infection of European minnows, *Phoxinus phoxinus*, with the cestode *L. intestinalis* affected their shoaling behaviour: infected minnows had larger nearest neighbour distance and more likely occupied peripheral shoal positions compared to uninfected minnows.

We did not observe differences in risk-taking behaviour between *S. solidus-*infected and uninfected sticklebacks before the bird attack. A possible explanation for this might be that the sticklebacks had not yet experienced predation (they grew up in our aquaria facility), they did not have a reason to avoid the potentially more ‘dangerous’ open water before the bird attack occurred. However, after the bird attack, all sticklebacks were aware of the presence of a predator and had to reconsider the risk-balancing trade-off [47,57] between foraging and staying in the ‘safe’ zone. We did not expose the sticklebacks to bird attacks repeatedly. Future research could use repeated simulated predation events to test if this amplifies or erodes differences in risk-taking between infected and uninfected individuals.

In natural situations, with real bird predators, behavioural changes of uninfected sticklebacks, as observed in the present study, could make them more prone to predation as well. This might not only have implications for the uninfected hosts, which follow the infected ones and become a bird's target, it might also increase the attractiveness of the habitat for fish-eating birds, in general. Higher life cycle completion rates of the parasites are a possible outcome if the bird predation rates on the parasite affected population increase due to elevated prey availability. Thus, the behavioural change of uninfected sticklebacks, as observed in the present study could benefit the fitness of *S. solidus*. On the other hand, uninfected sticklebacks would dilute the presence of *S. solidus* in the bird's prey, which might neutralize positive fitness effects for the parasite. In the wild, survivors of predation attacks might increase their cautiousness thus potentially readjust their quorum escape response according to infection intensity of the manipulative parasite and the predation pressure by its bird hosts.

## 5. Conclusion

To the best of our knowledge, the present study showed for the first time that a parasite with a complex life cycle indirectly manipulates the shoaling behaviour of uninfected individuals of its host species. Thereby the infection rate within a shoal was a major determinant for the occurrence of a manipulation of the behaviour of uninfected hosts. By manipulation of the uninfected individuals, a population as a whole might become more attractive to predators, thereby increasing the predation risk for all individuals in the shoal and potentially increasing the life cycle completion rates of the parasite. This result highlights the importance for increasing our knowledge on how extensive the influence of infected individuals is on the behaviours of uninfected individuals, engaged in social networks.

## Data accessibility

Data are deposited in the Dryad Digital Repository at https://doi.org/10.5061/dryad.46j2nj6 [58].
